# Point-of-care ultrasound may be useful for detecting pediatric intussusception at an early stage

**DOI:** 10.1186/s12887-020-02060-6

**Published:** 2020-04-13

**Authors:** Jeong-Yong Lee, Jung Heon Kim, Seung Jun Choi, Jong Seung Lee, Jeong-Min Ryu

**Affiliations:** 1 Department of Pediatrics, Asan Medical Center, University of Ulsan College of Medicine, 88 Olympic-ro 43-gil, Songpa-gu, Seoul, 05505 Republic of Korea; 2 Department of Emergency Medicine, Ajou University School of Medicine, Suwon, Republic of Korea; 3 Department of Emergency Medicine, Asan Medical Center, University of Ulsan College of Medicine, Seoul, Republic of Korea

**Keywords:** Child, Diagnosis, Emergency service, hospital, Intussusception, Point-of-care systems, Ultrasonography

## Abstract

**Background:**

This study aimed to verify the usefulness of point-of-care ultrasound (POCUS) performed by pediatric emergency physicians for detecting intussusception at an early stage.

**Methods:**

This retrospective study included 1-month- to 6-year-old children with clinically suspected intussusception, who underwent POCUS in the pediatric emergency department between December 2016 and February 2018. The criteria for performing POCUS were set to broader standards: presenting any one of intermittent abdominal pain/irritability or bloody stool, or ≥ 2 symptoms among nonspecific abdominal pain/irritability, abdominal mass/distension, vomiting, or lethargy. POCUS results were interpreted and categorized as “negative” or “suspicious,” and a radiologist performed confirmatory ultrasound in “suspicious” cases.

**Results:**

We analyzed 575 POCUS scans from 549 patients (mean age, 25.5 months). Among the 92 “suspicious” cases (16.0%), 70 (12.2%) were confirmed to have intussusception. POCUS showed 100% sensitivity, 95.6% specificity, and 97.8% accuracy. Patients with confirmed intussusception were mainly diagnosed in the early stages, with a mean symptom duration of 11.7 h, and most patients (97.1%) were treated successfully via air enema reduction. Compared to the non-intussusception group, the intussusception group had more intermittent abdominal pain (*P* < 0.001), but less vomiting (*P* = 0.001); the other clinical features showed no intergroup differences.

**Conclusion:**

POCUS performed using the criteria set to broader standards by pediatric emergency physicians may be useful for detecting intussusception at an early stage, which may present with obscure clinical symptoms.

## Background

Intussusception is a significant cause of intestinal obstruction in the pediatric population, and it necessitates a visit to the emergency department (ED) [1]. Intussusception is often alleviated by therapeutic air or liquid enema, but a delay in diagnosis may lead to intestinal gangrene, perforation, and peritonitis, which may require unexpected surgery [2]. Therefore, screening suspected cases of intussusception and detecting it at an early stage in the ED are important.

Detecting intussusception by evaluating the clinical features or plain abdominal radiographs may be challenging [3]. The classic triad of abdominal pain/irritability, a palpable sausage-shaped mass, and bloody stool occurs in less than 40% of cases of intussusception, and these are often indistinguishable from the symptoms of acute gastroenteritis [4]. The sensitivity of radiographs interpreted by pediatric emergency physicians was disappointingly low as 48% [5]. Although multiple views of abdominal plain radiographs interpreted by an experienced radiologist might be better [6]. In contrast, ultrasound can accurately diagnose intussusception with a high accuracy of 97.9% sensitivity and 97.8% specificity, and therefore is recommended as the diagnostic modality of choice [7]. However, requesting radiologist-performed ultrasound (RADUS) in all clinically suspected cases might be time-consuming and inefficient for the ED workflow [8].

Several studies have suggested that point-of-care ultrasound (POCUS) performed by pediatric emergency physicians could be a practical measure [8–10]. POCUS for detecting intussusception is relatively easy to learn and readily available in the ED [10–12]. The aim of this study was to verify the usefulness of POCUS performed by pediatric emergency physicians for detecting intussusception at an early stage.

## Methods

This retrospective study included 1-month- to 6-year-old children with clinically suspected ileocolic intussusception, who underwent POCUS performed by pediatric emergency physicians in the pediatric ED of a tertiary-care university-affiliated hospital between December 2016 and February 2018. Patient data were collected by reviewing the electronic medical records. The institutional review board approved this study and waived the requirement for informed consent.

## Pocus

The criteria for performing POCUS were set to broader standards to detect intussusception at an early stage wherein POCUS was performed in the presence of the following symptoms: any one of intermittent abdominal pain/irritability or bloody stool, otherwise at least two symptoms among nonspecific abdominal pain/irritability, abdominal mass/distension, vomiting, or lethargy. These criteria were modified more inclusively base on the diagnostic criteria (a proposal) in the Japanese guidelines for the management of intussusception in children given in 2011 [13]. Patients transferred from another hospital with a confirmed diagnosis of intussusception were excluded. POCUS was performed by one of seven pediatric emergency physicians. They completed a 4-h pediatric ultrasound course certified by the Korean Society of Pediatric Emergency Medicine, and had a mean experience of approximately 3 years (range, 0–6 years) of performing POCUS in the ED. All POCUS procedures were performed in a separate dedicated room by using the iE33 (Philips Ultrasound, Bothell, Washington) with a 3- to 11-MHz linear or 5- to 8-MHz curvilinear transducer. POCUS scans were interpreted and categorized as “negative” or “suspicious” for intussusception. A “negative” result on POCUS was strictly defined as absolutely no observable target or pseudokidney sign, whereas a “suspicious” result was defined as any presence of those signs or equivocal findings.

### Management of patients

Patients with “suspicious” POCUS results were treated using fluid replacement and were transferred for RADUS. In addition, the pediatric emergency physicians requested RADUS in a few cases of “negative” POCUS results for evaluating conditions other than ileocolic intussusception. Intussusceptions confirmed using RADUS were evaluated for reducibility, and the radiologists subsequently attempted air enema reduction. Patients with successfully reduced intussusceptions were discharged after approximately 6 h of observation in the ED for possible recurrence or other complications, whereas patients in whom more than two attempts of air enema reduction resulted in failure were considered for surgery. Patients in whom intussusceptions recurred after their discharge from the ED were counted separately. Patients requiring surgery or showing recurrent intussusception within 48 h were admitted to the hospital. Most of the patients with “negative” POCUS results were discharged safely after ensuring they or their parents had a full understanding of the symptoms that would necessitate a revisit to our ED.

### Data analysis

Patient data were categorized according to clinical features (age, sex, time of ED visit, previous ED visit within 24 h, duration of symptoms, and clinical symptoms), POCUS and RADUS (interpretations and time from ED arrival to performing POCUS or RADUS), and management outcomes (air enema reduction, surgery, recurrence within 48 h, admission, and ED observation time). The duration of symptoms was defined on the basis of the time of onset of abdominal pain/irritability (otherwise, lethargy or bloody stool) as determined by the parents; the time of onset was double-checked by a nurse as well as a physician with the time interval in all cases.

The POCUS results were analyzed using MedCalc version 18.11.6 (MedCalc software, Ostend, Belgium) and presented as 95% confidence intervals. Comparisons of clinical features between the intussusception and non-intussusception groups were analyzed using the χ^2^ test for categorical variables and *t* test for continuous variables by using IBM SPSS Statistics for Windows, Version 20.0 (IBM Corp., Armonk, New York). *P* < 0.05 was considered statistically significant.

## Results

### Patient characteristics

We analyzed 575 POCUS scans from 549 patients. The mean age of the patients was 25.5 ± 15.9 months (range, 1–81 months) and 297 patients (51.7%) were male. The mean duration of time from arrival at the ED to undergoing POCUS was 54.7 ± 74.7 min. Among 92 patients (16.0%) with “suspicious” POCUS results, 70 (12.2%) were confirmed to have intussusception using RADUS. These patients mainly had intussusception in the early stages, showing symptoms for a mean duration of 11.7 ± 15.6 h. Treatment of all patients with intussusception was attempted via air enema reduction, which was successful in 68 patients (97.1%). More than two attempts of air enema reduction resulted in failure in only 2 patients (one with suspected appendiceal intussusception requiring appendectomy and another with a delayed ED visit after 48 h of abdominal pain), and they underwent surgical reduction. Table 1 shows the patient characteristics, including time management in the ED.

**Table 1:** Patient characteristics

Characteristic	Value
Age, months, mean ± SD	25.5 ± 15.9
Sex, male, n (%)	297/575 (51.7%)
Duration of symptoms, hours, mean ± SD	9.9 ± 13.2
Door-to-POCUS time, minutes, mean ± SD	54.7 ± 74.7
ED length of stay, hours, mean ± SD	3.5 ± 3.0
Ileocolic intussusception, n (%)	70/575 (12.2%)
Successful air enema reduction, n (%)	68/70 (97.1%)
Surgical reduction, n (%)	2/70 (2.9%)
Recurrence within 48 h, n (%)	12/70 (17.1%)
Admission, n (%)	14/70 (20%)
Door-to-RADUS time, minutes, mean ± SD	72.2 ± 56.7
ED observation time after reduction, hours, mean ± SD	6.9 ± 2.9

*SD* Standard deviation, *POCUS* Point-of-care ultrasound, *ED* Emergency department, *RADUS* Radiologist-performed ultrasound

### POCUS performance

**Fig. 1:**
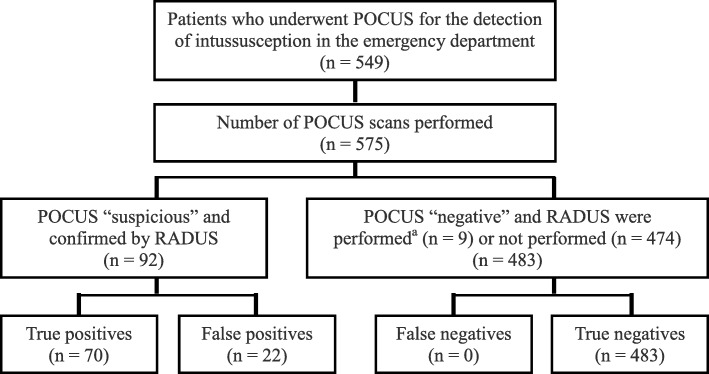
Flowchart showing the performance characteristics of point-of-care ultrasound (POCUS). ^a^ Radiologist-performed ultrasound (RADUS) was performed for evaluating conditions other than ileocolic intussusception

The performance characteristics of POCUS are presented as a flowchart in Fig. 1. Among the 92 patients with “suspicious” POCUS results, 22 were considered false positives, including 6 with an edematous ileocecal valve and 2 with small bowel intussusception. The remaining 483 patients with “negative” POCUS results were considered true negatives. Among them, RADUS was performed in 9 patients for evaluating conditions other than ileocolic intussusception (suspicious small bowel intussusception, unexplained ileus, or prominent intra-abdominal fluid collection), including 2 with small bowel intussusception and one with an intestinal duplication cyst, but no ileocolic intussusception. The remaining 474 patients with “negative” POCUS results, who did not undergo RADUS, were discharged safely from the ED, except for 10 patients who were admitted to the hospital for supportive care. Among the 402 patients (84.8%) who visited our hospital again, we could not find any records indicating diagnosis of intussusception from another hospital within 2 years after discharge from the ED. A total of 35 patients (7.4%) revisited within 48 h of ED discharge, and none of these patients were diagnosed with intussusception. The performance results of POCUS are summarized in Table 2.

**Table 2:** Performance results of point-of-care ultrasound

Indicator	Value (95% CI)
Sensitivity	100% (94.9%–100%)
Specificity	95.6% (93.5%–97.3%)
Accuracy	97.8% (96.3%–98.9%)
Positive predictive value	76.1% (67.9%–82.7%)
Negative predictive value	100% (100% − 100)
Positive likelihood ratio	23.0 (15.3–34.5)
Negative likelihood ratio	0 (0–0)

*CI* Confidence interval

### Clinical features

The common clinical symptoms of the enrolled patients were abdominal pain/irritability (87.5%), vomiting (52.7%), loose stool (20.2%), fever (19.8%), and bloody stool (15.3%). The intussusception group demonstrated abdominal pain/irritability (92.9%), vomiting (37.1%), and bloody stool (21.4%). Compared to the non-intussusception group, the intussusception group showed more intermittent abdominal pain/irritability (58.6% vs. 33.9%, *P* < 0.001), but significantly less vomiting (37.1% vs. 58.2%, *P* = 0.001). Other clinical features, including bloody stool, lethargy, ED visit during night-time (7 pm to 8 am), or duration of symptoms, were not significantly different between the two groups (Table 3).

**Table 3:** Comparison of clinical features between the intussusception and non-intussusception groups

	Intussusception group (***n*** = 70)	Non-intussusception group (***n*** = 505)	***P***
Age, mean ± SD, months	25.6 ± 16.8	25.5 ± 15.8	0.966
Sex, male, n (%)	41/70 (58.6%)	256/505 (50.7%)	0.216
Time of ED visit			
Night-time (7 pm − 8 am)	36/70 (51.4%)	311/505 (61.6%)	0.104
Day-time (8 am−7 pm)	34/70 (48.6%)	194/505 (38.4%)	
Previous ED visit within 24 h	7/70 (10%)	25/505 (5%)	0.084
Duration of symptoms, mean ± SD, hours	11.7 ± 15.6	9.7 ± 12.9	0.238
Abdominal pain/irritability	65/70 (92.9%)	438/505 (86.7%)	0.147
Intermittent	41/70 (58.6%)	171/505 (33.9%)	< 0.001
Nonspecific	24/70 (34.3%)	267/505 (52.9%)	0.004
Bloody stool	15/70 (21.4%)	73/505 (14.5%)	0.129
Abdominal mass/distension	10/70 (14.3%)	64/505 (12.7%)	0.706
Vomiting	26/70 (37.1%)	294/505 (58.2%)	0.001
Lethargy	5/70 (7.1%)	51/505 (10.1%)	0.434
Fever	13/70 (18.6%)	101/505 (20.0%)	0.779
Loose stool	10/70 (14.3%)	106/505 (21.0%)	0.190

*SD* Standard deviation, *ED* Emergency department

## Discussion

This study verified the usefulness of POCUS performed by pediatric emergency physicians by applying criteria set to broader standards for detecting intussusception at an early stage. Our results demonstrated a relatively short symptom duration of 11.7 h in intussusception cases, excellent performance outcome of POCUS, and the limitations of clinical features for distinguishing the intussusception and non-intussusception groups.

In this study, POCUS performed by pediatric emergency physicians seemed highly reliable (sensitivity, 100%; specificity, 95.6%; and accuracy, 97.8%) and useful (positive likelihood ratio, 23.0; and negative likelihood ratio, 0) in detecting intussusception. A previous study similarly showed a high degree of accuracy with 100% sensitivity and 94% specificity, although a relatively small number of 49 patients were enrolled [9]. Our promising results could support the usage of POCUS for intussusception in the pediatric ED, especially with the limited resources of radiologists available.

The positive predictive value of 76.1% in our study (22 patients of false positives among 92 patients with “suspicious” POCUS results) seems to be low, but it could be explained. First, these false positives included 6 cases of edematous ileocecal valves, which were presumed as spontaneously reduced intussusceptions between performing POCUS and RADUS. Secondly, we interpreted all the uncertain cases from POCUS as “suspicious” results, even though the possibility of intussusception was low. In the ED setting, a missed diagnosis of intussusception possibly leads to serious consequences; therefore, the interpretation criteria of POCUS should be strict regarding the exclusion of intussusception as the diagnosis. Then it would be preferable to request RADUS for the cases of “suspicious” POCUS results to confirm the diagnosis, as well as to evaluate reducibility or the possible presence of a pathologic lead point. Therefore, we considered it more important to reduce false negatives than false positives in this study, since performing POCUS was intended to screen suspected intussusception and to rule out non-intussusception cases.

Proactively performing POCUS by applying criteria set to broader standards seems to facilitate the detection of intussusception at an early stage. Accordingly, this study aimed to perform POCUS by applying criteria set to broader standards in patients presenting any one of intermittent abdominal pain/irritability or bloody stool, or at least two symptoms among nonspecific abdominal pain/irritability, abdominal mass/distension, vomiting, or lethargy. Consequently, the enrolled patients with intussusception were considered to be in the early stage, which presented symptoms for a much shorter mean duration of 11.7 h instead of a duration of over 18.5 h reported in previous studies [14–16]. The favorable treatment outcome in 97.1% of patients with successful air enema reduction also indirectly indicates that the patients were in early stages of intussusception; only 2 patients required surgical reduction. Moreover, the intussusception group in this study presented a lower prevalence of vomiting (37.1%) and bloody stool (21.4%) than did those in previous studies, which reported vomiting in 85% and bloody stool in up to 65% of patients [17]. According to the clinical course of intussusception, as intestinal obstruction progresses, abdominal pain appears first, followed by vomiting and bloody stool [4, 13]. Thus, our findings indicated that most patients with intussusception were in the early stage and had not yet developed vomiting.

Compared with the non-intussusception group, the intussusception group presented more intermittent abdominal pain (*P* < 0.001), but less vomiting (*P* = 0.001); however, the other clinical features were not significantly different. Only intermittent abdominal pain/irritability (58.6%) seems helpful in distinguishing intussusception in the early stages in a clinical setting, and this may suggest that detecting intussusception would still be challenging without performing POCUS.

This study has several limitations owing to its retrospective, single-center design. Most of the patients with “negative” POCUS results were not confirmed to have intussusception using RADUS; thus the possibility of false-negative results could exist. However, we strictly ruled out patients without intussusception, and also carefully reviewed their follow-up medical records in 84.8%; none of them were proven to have intussusception within 48 h of ED discharge. Defining the onset of intussusception based on the duration of symptoms determined by the parents might be incorrect, although we double-checked the presumed time. Furthermore, our medical resources other than POCUS may have affected the treatment outcomes, which might potentially limit the generalizability of the findings of our study. We also did not consider the individual POCUS experience of pediatric emergency physicians, cost-effectiveness of POCUS, and satisfaction of the children or parents. Further prospective, multicenter studies are required to address these issues.

## Conclusions

POCUS may be performed by pediatric emergency physicians to detect intussusception. Furthermore, performing POCUS by applying criteria set to broader standards in the ED could help detect intussusception at an early stage, which may present with obscure clinical symptoms.

## Data Availability

The dataset used and analyzed in this study is available from the corresponding author on request.

## Abbreviations

ED: Emergency department
POCUS: Point-of-care ultrasound
RADUS: Radiologist-performed ultrasound
